# Case of Pneumonia

**Published:** 1901-09

**Authors:** 


16th, 6 A.M., temperature 100°.

### Case of Pneumonia.

A chestnut gelding, eight years old, arrived from Norfolk, Va., and caught a severe chill by being put near the engine-room.

*Symptoms.* Rapid breathing, flapping of nostrils, slight discharge. Temperature, 105° F.

9.15 A.M. ℞.—Acetanilid, ʒij.; qui. sulph., ʒj.; vaseline put upon tongue.

1 P.M., temperature $104\frac{3}{5}$° F. ℞.—Acetanilid, ʒj.; sodæ salicyl., ʒss.; vaseline.

3.15 P.M., temperature 104°. ℞.—Quin. sulph., ʒj.; pot. bicarb., ʒij.

7 P.M. Above repeated.

9 P.M., temperature 103° F. ℞.—Put tr. aconite and pot. nit. in bucket of water.

9th, 6 A.M., temperature 106$\frac{2}{5}$°. ℞.—Acetanilid, sodæ salicyl., of each ʒij.; proof rum, ℥ij.; aqua; put on rug, but skin inactive; partaken of bran mash in which ℥ij. Epsom salts were put.

8 A.M., temperature 104°.

11 A.M., temperature 103$\frac{2}{5}$°. ℞.—Acetanilid, sodæ salicyl., of each ʒij.; proof rum, ℥ij.; aqua, q. s. pint bottle.

1 P.M., temperature 103$\frac{1}{5}$°.

2 P.M. ℞.—Acetanilid, ʒj.; quinine sulph., ʒj.; vaseline. Cough troublesome and painful.

5 P.M., temperature 105°. Above repeated.

9 P.M., temperature 105°. ℞.—Liq. amm. acet. C.°, ʒij.; sprts. æth. nit., ℥j.; tr. aconite, ♏xx.; aqua q. s. pint.

Aconite and pot. nit. put in drinking water.

10th, 6 A.M., temperature 105$\frac{2}{5}$°.

8 A.M., temperature 106$\frac{2}{5}$°. ℞.—Sprts. æth. nit., ℥j.; liq. amm. acet. C.°, ʒij.; tr. camph. C.°, ʒiv.; tr. aconite, ♏xx.; acid. carbolic., ♏xx. in ℥xij. ol. lini.; clipped sides, applied tr. amm. comp. and rugged up. There were no abnormal sounds emitted by the lungs; very little discharge from the nostrils, but cough sonorous and mucus emitted from the mouth during the act.

10 A.M., temperature 107° F. ℞.—Acetanilid, ʒij.; whiskey, ℥iv.; tr. scillæ, ʒij.; tr. camph. C.°, ʒvj.; aqua, q. s. pint bottle.

12.30 P.M., temperature 105$\frac{2}{5}$°; eats hay.

2 P.M., temperature 106°; ℥iv. whiskey in water.

4 P.M., temperature 105$\frac{2}{5}$°; ℥iv. whiskey in water.

6 P.M., temperature 105.

9 P.M., temperature 105°. ℞.—Whiskey, ℥iv.; acetanilid, ʒij.

11th, 6 A.M., temperature 103$\frac{2}{5}$°. Eaten a bran mash; looks more cheerful, and picking at hay.

11 A.M., temperature 105°; breathing quickly. ℞.—Whiskey, ℥ij.; tr. scillæ, ʒij.; tr. camphor. C.°, ʒiv.; acetanilid, ʒiss.; sodæ salicyl., ʒj.; acid. carb., ♏xij.; aqua, q. s. pint bottle.

3.30 P.M., temperature, 104$\frac{1}{5}$°. ℞.—Whiskey, ℥ij.; tr. camph. C.°, ʒiv.; liq. amm. acet., C.°, ʒj.; aqua, ℥j.

7 P.M., temperature 106° F.

9 P.M., temperature 105° F. ℞.—Liq. amm. acet. C.°, ʒij.; sprts. æth. nit., ʒj. ; tr. aconite, ♏xx. ; aqua, q. s. ℥xiii. Forty minims tr. aconite and ʒiv. pot. nit. put into drinking water.

12th, 6 A.M., temperature 104°. ℞.—Acetanilid, ʒij. ; quinine sulph., ʒj. ; vaseline.

8 A.M., temperature 105° F. One loose motion. Cough rasping and sonorous; no discharge from nostrils; pulse and breathing 60.

1 P.M., temperature $104\frac{2}{5}$°. ℞.—Acetanilid, ʒij. ; whiskey, ℥iv. ; tr. camph. C.°, ʒiv. ; glycerin, ℥j. ; aqua, q. s. ℥xiij.

3.45 P.M., temperature 103° F.

6 P.M., temperature 103° F. ℞.—Acetanilid, ʒij. ; whiskey, ℥iv. ; tr. camph. C.°, ʒiv. ; glycerin, ℥j. ; aqua, q. s. ℥xiij.

10 P.M., temperature 103° F. ℞.—Liq. amm. acet. C.°, ʒj. ; sprts. æth. nit., ʒiv. ; tr. aconite, ♏xx. ; acid. carbolic., ♏x. ; aqua, ℥x.

13th, 7 A.M., temperature 104° F. ℞.—Whiskey, ℥ij. ; tr. camph. C.°, ʒiv. ; sodæ salicyl., ʒj. ; aqua, q. s. ℥xiij. Two good motions, healthy in appearance and consistency; ate bran mash, and, although looks weak, still is bright. Cough rasping and troublesome. Cough mixture of pot. chlor. and quin. sulph., vaseline, ext. bellad. put at intervals on tongue ; eating hay and grass.

12 noon, temperature 105° ℞.—Liq. amm. acet. C.°, ʒij. ; sprts. æth. nit., ʒiv. ; tr. camph. C.°, ʒiv. ; tr. aconite, ♏x.; aqua, q. s. ℥xiij.

4 P.M., temperature, 104°.

6 P.M., temperature 105°. Above dose repeated.

10 P.M., temperature 105°. ℞.—Quinine sulph., ʒj. ; acetanilid, ʒj. ; vaseline.

14th, 6 A.M., temperature $103\frac{2}{5}$°. ℞.—Quinine sulph., ʒj. ; acetanilid, ʒj. ; vaseline. Appetite improved; appearance brighter, and cough less rasping.

10 A.M., temperature 103°.

2 P.M., temperature $103\frac{2}{5}$°.

4 P.M., temperature 104°. Above repeated.

15th, 6 A.M., temperature 102°.

8 A.M., temperature 102°. ℞.—Sprts. chlor., ʒiv. ; tr. camph. C.°, ʒiv. ; tr. cinchon, ʒij. ; tr. scillæ, ʒij. ; aqua, q. s. ℥xiij.

10 A.M., temperature 102°.

12 noon, temperature $102\frac{3}{5}$°. ℞.—Quin. sulph., ʒj. ; acetanilid, ʒj. ; vaseline.

6 P.M., above repeated.

16th, 6 A.M., temperature $105\frac{2}{5}°$.

12 noon, temperature $105\frac{2}{6}°$.

4 P.M., temperature $100\frac{4}{5}°$. Above repeated.

17th, 6 A.M., temperature $100\frac{4}{5}°$. From hence, cough mixture put upon tongue twice daily. Animal discharged, cured, on the 26th.

